# 

**Published:** 1814-11

**Authors:** 


### MONTHLY CATALOGUE OF MEDICAL BOOKS.

A PRACTICAL TREATISE on Porrigo, or Scald Head; and on Impetigo, the humid or running Tetter: with coloured Engravings, illustrative of the Diseases. By the late Robert Willan, M.D. F.R.S. and F.A.S. Edited by Ashby Smith, Member of the Royal College of Surgeons. 4to. 12s.

Cases of Tetanus and Rabies Contagiosa, or Canine Hydrophobia; with Remarks, chiefly intended to ascertain the Characteristic Symptoms of the latter Disease in Man and certain Brutes, and to point out the most effectual Means of Prevention. By Caleb Hillier Parry, M.D. F.R.S. 8vo.

A Synopsis of the various Kinds of Difficult Parturition; with Practical Remarks on the Management of Labour. By Samuel Merriman, M.D. Teacher of Midwifery. Second Edition, with considerable Additions. 12mo. boards, 6s.

Observations on Adhesion; with Two Cases, demonstrative of the Powers of Nature to reunite Parts which have been by Accident totally separated from the Animal System. By William Balfour, M.D. 8vo. sewed, 1s. 6d.

### TO CORRESPONDENTS.

*Several Communications have arrived too late for publication this Month, but will appear in our next Number.*

